# Understanding anxiety disorders among Iranian mothers and their children exposed to conflict using an extended transactional model of stress and coping

**DOI:** 10.3389/fpsyt.2026.1839849

**Published:** 2026-10-16

**Authors:** Nie Ni, Zahra Osati, Zeinab Zaremohzzabieh, Seyedali Ahrari, Haslinda Abdullah

**Affiliations:** 1 Department of Educational Science, Guangxi Minzu Normal University, Chongzuo, China; 2 Faculty of Woman and Family, University of Religions and Denominations, Qom, Iran; 3 Women and Family Studies Research Center, University of Religions and Denominations, Qom, Iran; 4 Institute for Social Science Studies, Universiti Putra Malaysia, Serdang, Selangor, Malaysia

**Keywords:** child anxiety, cognitive appraisal, coping strategies, extended transactional model, maternal anxiety, religiosity, social support, war-related stressors

## Abstract

**Objective:**

The psychological impact of war on families is profound. However, the mechanisms linking war-related stressors to anxiety among mothers and their children remain insufficiently understood, particularly in Middle Eastern contexts. Grounded in an extended transactional model of stress and coping, this study examined the direct and indirect relationships between war-related stressors, cognitive appraisal, coping strategies, and anxiety among Iranian mothers and their children following a recent military conflict. The study also investigated the mediating roles of appraisal and coping processes and the moderating roles of religiosity and social support.

**Method:**

A cross-sectional design was employed approximately three months after a major military escalation. A stratified proportional sample of 343 Iranian mothers (aged 24 years and older) with at least one child aged 5–10 years living in conflict-affected areas completed validated self-report measures. Data were analyzed using partial least squares structural equation modeling (PLS-SEM).

**Results:**

The extends model explained substantial variance in maternal anxiety (*R*^²^ = .80) and child anxiety (*R*^²^ = .72). War-related stressors were significantly and positively associated with both maternal anxiety and child anxiety. In addition, maternal anxiety was significantly associated with child anxiety. Challenge appraisal, problem-focused coping, and cognitive restructuring mediated the relationship between war-related stressors and maternal anxiety. Emotion-focused coping positively mediated the relationship between war-related stressors and child anxiety. Religiosity and social support were found to buffer the association between war-related stressors and maternal anxiety, such that higher levels of religiosity and social support weakened the positive relationship between war-related stressors and anxiety among mothers.

**Conclusions:**

This study extends the transactional model of stress and coping by supporting an integrated framework within a conflict-affected Middle Eastern population. The findings indicate distinct pathways linking war-related stressors to maternal and child anxiety and highlight the importance of interventions that address cognitive appraisal, strengthen adaptive coping strategies, and mobilize spiritual and social resources in conflict-affected families.

## 1 Introduction

Armed conflicts are among the most severe collective stressors affecting family well-being, with mothers and children particularly vulnerable to prolonged uncertainty, fear, displacement, and disruption of daily life. Exposure to war-related stressors has been associated with elevated anxiety and other psychological difficulties. Studies have reported maternal anxiety prevalence rates ranging from 38.1% to 61%, depending on the conflict context and assessment methods (1, 2). Children living in conflict-affected settings similarly experience substantial psychological difficulties, with reported rates ranging from 6% for post-traumatic stress disorder among preschool children in Gaza to 67% for clinical anxiety among refugee children (3). These findings demonstrate the considerable psychological burden of armed conflict and highlight the importance of understanding factors associated with anxiety among mothers and their children.

Research further suggests that maternal and child psychological outcomes may be interconnected during and following armed conflict. Studies conducted during active conflict or shortly after its onset have reported associations between maternal anxiety and child emotional distress (1, 2). Possible mechanisms include emotional contagion, maternal emotion regulation, and changes in caregiving behavior (4, 5). Physiological processes may also contribute, as synchrony in stress-related biomarkers has been observed between mothers and children (6). However, findings are not consistent across contexts. For example, a longitudinal study of Kosovar families conducted 11 years after the war found no significant association between maternal and child anxiety symptoms (2). These differences suggest that the relationship between maternal and child anxiety may depend on the timing, intensity, and broader context of conflict exposure.

The psychological consequences of war are also influenced by the type and intensity of stressors experienced. Children may encounter repeated threats, violence, displacement, separation from caregivers, and other distressing experiences. Studies in Gaza have documented extensive exposure to shelling, drone activity, and other traumatic experiences among children (3), while similar patterns have been reported among Middle Eastern refugee populations (7). Direct exposure to violence, parental separation, and adverse family experiences are important risk factors for psychological distress (8). Nevertheless, individuals exposed to similar stressors may experience different psychological outcomes, highlighting the importance of examining the processes through which war-related stressors influence anxiety.

The Transactional Model of Stress and Coping provides a useful framework for understanding these processes. According to Lazarus and Folkman, psychological stress arises from the interaction between environmental demands and an individual’s appraisal of those demands and available coping resources. Cognitive appraisal determines whether an event is perceived as threatening, challenging, or manageable, whereas coping involves cognitive and behavioral efforts to manage stressful demands (3, 4). These processes may influence whether exposure to war-related stressors results in psychological distress or adaptation. Importantly, appraisal and coping occur within broader social and cultural contexts. Religiosity may provide meaning, spiritual comfort, and guidance for coping with adversity (9–11), while social support may strengthen perceived coping resources and reduce feelings of isolation (12).

Despite growing recognition of the psychological consequences of armed conflict, several gaps remain. Existing research has examined war exposure, psychological distress, coping strategies, cognitive appraisal, and social or family resources, but these processes have often been investigated separately or in limited combinations (13–15). Rizzi et al. (15), for example, identified problem-focused, emotion-focused, avoidance, faith-based, and belonging-related coping strategies among Ukrainian refugees and internally displaced persons, while also highlighting the importance of social connections and protective factors. More recently, Figueiredo and Petravičiūtė (16) reviewed coping strategies among forcibly displaced populations and emphasized the need to better understand how coping relates to psychological outcomes. In addition, Buchnik-Atzil et al. (2) found a positive association between maternal anxiety and child emotional distress during wartime and demonstrated that family-level resources could buffer this association. However, comparatively little research has integrated war-related stressors, cognitive appraisal, coping strategies, anxiety, and sociocultural resources within the same mother–child framework. Therefore, the present study examines the relationships among these factors within a Middle Eastern context, focusing on the mediating roles of appraisal and coping and the moderating roles of religiosity and social support.

## 2 Theoretical framework and hypotheses

The present study is grounded in the Transactional Model of Stress and Coping developed by Lazarus and Folkman (6). The model conceptualizes stress as a dynamic transaction between an individual and the environment rather than as a direct consequence of an external event. An event becomes stressful when it is appraised as significant to well-being and as taxing or exceeding available coping resources. Thus, similar stressful experiences may produce different psychological outcomes depending on appraisal and coping resources. Cognitive appraisal consists of primary and secondary appraisal. Primary appraisal involves evaluating whether an event is irrelevant, benign-positive, or stressful. Stressful events may subsequently be appraised as harm/loss, threat, or challenge. Harm/loss concerns damage that has already occurred, threat concerns anticipated harm, and challenge reflects the perception that a demanding situation may provide opportunities for growth or mastery. Secondary appraisal concerns the evaluation of available coping resources and options for managing the situation (6).

Coping refers to cognitive and behavioral efforts to manage demands appraised as stressful. It is a dynamic process that varies according to the stressor, appraisal, and available resources. Problem-focused coping involves attempts to manage the source of stress, whereas emotion-focused coping involves regulating emotional responses to the stressor. Appraisal and coping continuously influence one another and help explain why similar stressful experiences may result in different psychological outcomes. Challenge and threat appraisals may lead to different coping and emotional pathways. Challenge appraisal is generally associated with perceptions of adequate resources and greater task-focused coping, whereas threat appraisal reflects perceptions that demands exceed available resources and may be associated with greater distress (11, 17, 18). These distinctions are particularly relevant to war-related stressors, where violence, insecurity, displacement, loss, and uncertainty may be interpreted differently depending on perceived resources.

The model also allows consideration of broader protective resources. Problem-focused coping and cognitive restructuring may help individuals manage stressful demands or reinterpret their meaning, whereas avoidance and disengagement may be less adaptive when they prevent effective coping. Social support and religiosity may additionally provide emotional, cognitive, and social resources for managing adversity. Accordingly, the transactional framework provides the theoretical basis for examining the proposed mediating and moderating pathways.

War-related stressors include exposure to violence, insecurity, loss, displacement, family disruption, and ongoing daily stressors associated with armed conflict (19, 20). Unlike a single stressful event, war may involve repeated and prolonged exposure to threatening circumstances. Research has shown that war exposure and displacement-related stressors are associated with substantial psychological difficulties among conflict-affected populations (21–23). Mothers may be particularly vulnerable because they often experience concerns regarding both their own safety and that of their children. Miller et al. (24), for example, found that war-related events and daily displacement stressors were associated with psychological difficulties among Syrian refugee mothers. Similarly, reviews of conflict-affected populations have documented elevated anxiety, depression, and post-traumatic stress following exposure to war and displacement (22, 23). Therefore:

H_1_. War-related stressors will be positively related to maternal anxiety.

Children may similarly experience psychological difficulties following exposure to violence, insecurity, displacement, loss, and family disruption. Research has consistently documented anxiety, post-traumatic stress, depression, and behavioral difficulties among conflict-affected children (22, 25, 26). Kadir et al. (19) further emphasized the multiple and interconnected adversities experienced by children affected by armed conflict. Therefore:

H_2_. War-related stressors will be positively related to child anxiety.

Within the Transactional Model, appraisal determines how individuals interpret stressful encounters. Threat appraisal occurs when anticipated harm or loss is perceived, whereas challenge appraisal reflects the perception that a demanding situation may be manageable and provide opportunities for mastery (6, 7). Threat appraisal has been associated with anxiety and emotional distress, while challenge appraisal is generally associated with perceptions of sufficient coping resources (17, 18). Recent research has also linked threat appraisal with pediatric anxiety, supporting its relevance to children’s responses to stressful experiences (27). In war contexts, children may perceive violence, uncertainty, loss, and insecurity as threatening, particularly when they perceive limited control over these circumstances. Threat appraisal may therefore constitute a pathway through which war-related stressors contribute to child anxiety:

H_3_. Threat appraisal will mediate the relationship between war-related stressors and child anxiety.

Conversely, mothers who perceive stressful circumstances as challenges may evaluate themselves as having greater resources or capacity to manage demands. Challenge appraisal may encourage more active coping and reduce the perception that stressful circumstances are overwhelming [Seery, 2013 (17); Wang et al., 2016 (18)]. Thus:

H_4_. Challenge appraisal will mediate the relationship between war-related stressors and maternal anxiety.

Coping Mechanism represents the cognitive and behavioral efforts used to manage stressful demands. Recent reviews of conflict-affected and displaced populations identify problem-focused, emotion-focused, avoidance, faith-based, and social-support-related coping strategies as important responses to war and displacement (15, 16). Problem-focused coping includes planning, problem solving, information seeking, and practical action. Such strategies may be particularly relevant when individuals perceive some degree of control over stressful circumstances. Evidence among conflict-affected populations suggests that problem-focused coping is commonly used and may be associated with better psychological adjustment in some contexts, although its effects vary according to the nature of the stressor (15, 28). Therefore:

H_5_. Problem-focused coping will mediate the relationship between war-related stressors and maternal anxiety.

Cognitive restructuring involves changing or reframing the interpretation of a stressful situation. It is consistent with the transactional model because reinterpretation may alter the appraisal and emotional meaning of stressful experiences. Positive cognitive restructuring has been identified as a coping strategy among conflict-affected populations (15), while Carlsson et al. (29) examined cognitive restructuring among trauma-affected refugees. Thus, cognitive restructuring may represent a pathway through which mothers respond psychologically to war-related stressors:

H_6_. Cognitive restructuring will mediate the relationship between war-related stressors and maternal anxiety.

Emotion-focused coping involves attempts to regulate emotional responses to stress, including emotional expression, acceptance, distraction, or seeking emotional support. Such coping should not automatically be considered maladaptive because its effectiveness depends on the controllability and persistence of the stressor. Recent research indicates that emotion-focused coping is common among forcibly displaced populations, although its relationship with psychological distress varies across contexts (15, 16). Among children exposed to ongoing warfare, coping strategies may be particularly important because children have limited control over war-related circumstances. Therefore:

H_7_. Emotion-focused coping will mediate the relationship between war-related stressors and child anxiety.

Maternal anxiety may be associated with child anxiety through shared environmental exposure, parenting processes, maternal emotional regulation, and family interactions. Kerns et al. (30) found that maternal anxiety was associated with processes related to children’s anxiety, while more recent wartime research found a positive association between maternal anxiety and child emotional distress among families exposed to daily missile attacks. A broader literature also suggests associations between parental anxiety and offspring emotional outcomes, although findings vary according to developmental stage and context (31, 32). In war settings, mothers and children may experience the same threats, uncertainty, displacement, and disruption of family life. Maternal anxiety and child anxiety may therefore co-occur through shared exposure and reciprocal family processes rather than representing simple one-directional transmission. Therefore:

H8. Maternal anxiety will be positively related to child anxiety.

Religiosity may function as a psychological and social resource during periods of severe stress. Religious beliefs and practices may provide meaning, hope, spiritual comfort, and guidance for coping with circumstances that are difficult to control. Recent research among Ukrainian refugees and internally displaced persons identified faith-based coping as one of the major coping categories following war-related displacement (15). Similarly, Pertek (33) found that displaced women used religious resources, including prayer, spiritual practices, and religious meaning-making, to cope with traumatic experiences and displacement-related stressors. Within the transactional framework, religiosity may influence how mothers appraise and manage war-related stressors by providing additional cognitive and emotional resources. However, the effects of religiosity may depend on how religious beliefs are used, as positive and negative forms of religious coping can have different psychological consequences. Thus, religiosity may alter the strength of the association between war-related stressors and maternal anxiety:

H_9_. Religiosity will moderate the relationship between war-related stressors and maternal anxiety.

Social support may strengthen perceived coping resources and reduce the extent to which stressful demands are experienced as overwhelming. Support from parents, partners, family members, friends, peers, and community networks may provide emotional, practical, and informational resources during periods of conflict and displacement. Research among Ukrainian refugees and internally displaced persons has highlighted social connections and inclusive community interventions as important protective factors for psychological well-being (15). For children, supportive relationships may provide emotional security and assist in regulating distress following exposure to war-related stressors (34) For mothers, social support may reduce perceived demands associated with concerns about safety, caregiving, displacement, and family disruption. Recent wartime research also demonstrates that family-level resources can weaken associations between maternal anxiety and child emotional distress (2). Therefore, social support may buffer the psychological effects of war-related stressors for both children and mothers:

H_10_. Social support will moderate the relationship between war-related stressors and child anxiety.

H_11_. Social support will moderate the relationship between war-related stressors and maternal anxiety.

The study model and hypotheses based on the previous review are depicted in **Figure 1**.

**Figure 1:**
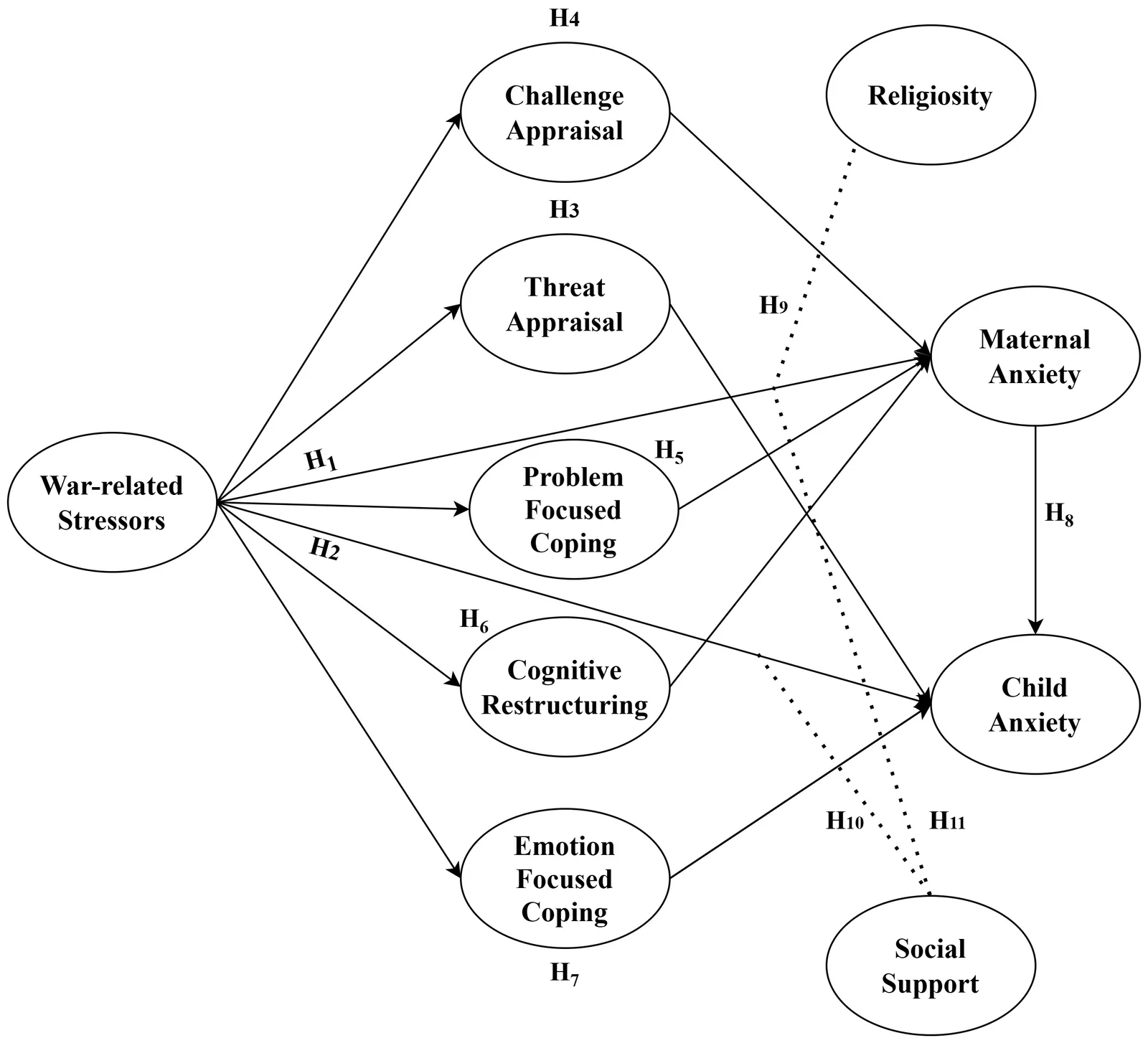
Study conceptual framework.

## 3 Materials and methods

### 3.1 Participants

This study employed a cross-sectional predictive design to examine anxiety among Iranian mothers of children exposed to the June 2025 Iran–Israel military conflict, which began on 13 June 2025 and continued for 12 days until a ceasefire was announced on 24 June 2025. Data were collected approximately three months after the cessation of the conflict to evaluate post-war cognitive appraisal processes, coping strategies, and anxiety symptoms within the framework of the extended transactional model of stress and coping.

### 3.2 Sampling and recruitment

A stratified proportional sampling design was implemented to ensure representation across geographic regions and displacement status following the June 2025 Iran–Israel military conflict, which began on 13 June 2025 and continued for 12 days until a ceasefire was announced on 24 June 2025. Population distribution data obtained from municipal and provincial administrative records were used to determine proportional allocation across strata. Stratification was conducted based on (a) geographic location (Tehran vs. selected provincial regions affected by the conflict) and (b) displacement status (displaced vs. non-displaced households). Within each stratum, participants were recruited through community-based outreach methods, including collaboration with local health centers, schools, women’s community organizations, and temporary housing facilities serving displaced families. This approach was designed to enhance representativeness and ensure access to both displaced and non-displaced mothers.

Inclusion criteria were defined as follows: participants were Iranian mothers aged 24 years or older who had at least one child aged 5 to 10 years residing in the household during the war period and who were living in a conflict-affected area at that time. The age range of 5 to 10 years corresponds to middle childhood, a developmental period characterized by increasing cognitive and emotional regulation capacities and relatively stable caregiving demands compared to early childhood. Participants were additionally required to be able to read and understand Persian to ensure independent completion of the questionnaires. Exclusion criteria included severe cognitive impairment that prevented accurate questionnaire completion and current inpatient psychiatric hospitalization at the time of data collection. Mothers with children under 5 years of age were excluded to maintain psychological homogeneity, as early childhood caregiving demands may introduce additional variability in stress and coping processes during conflict exposure.

An *a priori* power analysis was conducted using conventional parameters (α = .05, power = .80). Results indicated that a minimum sample size of approximately 300 participants would be required to detect medium effect sizes in multiple regression analyses with up to 10 predictors. The final sample of 343 participants exceeded this threshold, thereby ensuring adequate statistical power, improving parameter stability, and reducing the risk of Type II error.

All participants were fully informed about the study’s aims and procedures and provided written (or electronic) informed consent before participation. Confidentiality, anonymity, and data protection were strictly maintained, and participation was entirely voluntary, with the option to withdraw at any time without consequences. This study was conducted in accordance with the ethical standards of the institutional research committees, the Declaration of Helsinki (1964), and its later amendments or comparable ethical standards.

### 3.3 Procedure

Recruitment occurred between September and October 2025, approximately three to four months after the cessation of the June 2025 Iran–Israel military conflict, which began on 13 June 2025 and continued for 12 days until a ceasefire was announced on 24 June 2025. Potential participants were informed about the study’s objectives, the voluntary nature of participation, confidentiality safeguards, and their right to withdraw at any time without penalty. Written informed consent was obtained prior to participation. For displaced mothers residing in temporary housing, recruitment and data collection were conducted in coordination with local support agencies to ensure safety, privacy, and psychological sensitivity. Participants completed the survey either in supervised in-person sessions at community centers or health clinics, or in structured small-group sessions held in secure and accessible locations. The average completion time ranged from 25 to 35 minutes.

To minimize response bias and protect participant well-being, standardized instructions were provided to all participants. Research staff were trained in trauma-informed data collection procedures. Upon completion of the survey, participants were provided with contact information for brief psychological support services, should distress arise following participation. Data were collected at a single time point using self-administered structured questionnaires in Persian.

### 3.4 Measures

All measures were administered in Persian using standardized forward-backward translation procedures to ensure linguistic and conceptual equivalence. Demographic information, including age, education, occupation, marital status, family size, number of children, and displacement status, was collected using a structured questionnaire. The full set of items for all measures is provided in the **Supplementary Material**.

#### 3.4.1 War-related stressors

Exposure to war-related stressors was assessed using a structured self-report checklist adapted from prior conflict and trauma research (35–37). The scale assessed direct and indirect exposure to events such as displacement, loss of family members, destruction of property, witnessing violence, and deprivation of basic needs. The scale consists of 15 items. Participants rated the frequency or severity of each stressor on a five-point Likert scale ranging from 1 (not at all) to 5 (extremely frequent/severe). A total war stressor score was calculated by summing responses, with higher scores reflecting greater cumulative exposure. The measure demonstrated good internal consistency in the present sample (*α* = .86).

#### 3.4.2 Cognitive appraisal

Cognitive appraisal was assessed using the Revised Stress Appraisal Measure (38), selected because its items are distinct from measures of hardiness and coping. The scale comprises 12 items measuring two appraisal dimensions: threat appraisal and challenge appraisal. Participants rated each item on a four-point Likert scale ranging from 0 (*not at all*) to 3 (*very much*). A sample item for challenge appraisal is “*I felt I had the skills necessary to overcome the stressful event*,” and for threat appraisal, “*I felt this stressful event would have a negative impact on me*.” In the present study, internal consistency was acceptable for both subscales (threat appraisal *α* = .82; challenge appraisal *α* = .87).

#### 3.4.3 Coping strategies

Coping strategies were assessed using the Brief COPE (39), a 28-item self-report measure of coping responses. Participants rated each item on a four-point Likert scale ranging from 0 (*not at all*) to 3 (*very much*). Consistent with the theoretical framework of Carver et al. (40), a four-factor structure was employed: problem-focused coping (e.g., “*I took action to try to make the situation better*”; *α* = .83), cognitive restructuring (e.g., “*I learned to live with it*”; *α* = .74), social support (e.g., “*I tried to get advice or help from other people about what to do*”; *α* = .74), and emotion-focused coping (e.g., “*I gave up trying to deal with it*”; *α* = .70). Subscale scores were calculated by averaging relevant items.

#### 3.4.4 Religious coping

Religiosity was assessed using a multi-item scale to ensure adequate psychometric coverage of the construct. Selected items from the religious coping subscale of the Brief COPE (39) were supplemented with culturally adapted items reflecting Islamic practices and local Iranian traditions.The scale consists of 10 items. Items assessed engagement in practices such as prayer, fasting, Quranic recitation, and seeking guidance from religious leaders. Participants rated the frequency of each practice on a five-point Likert scale ranging from 1 (never) to 5 (very often). This approach aligns with culturally responsive research practices emphasizing contextual validity (41, 42). Internal consistency for the religious coping scale in the present sample was high (*α* = .91).

#### 3.4.5 Maternal anxiety

Maternal anxiety symptoms were assessed using the Generalized Anxiety Disorder-7 (GAD-7) (43), a widely used screening tool for generalized anxiety disorder. The scale consists of seven items evaluating the frequency of anxiety symptoms over the past two weeks (e.g., excessive worry, difficulty relaxing). Items were rated on a four-point Likert scale ranging from 0 (not at all) to 3 (nearly every day). Total scores were calculated by summing all items, with higher scores indicating greater anxiety severity. In the present study, the GAD-7 showed excellent internal consistency (*α* = .93).

#### 3.4.6 Child anxiety

Child anxiety symptoms were assessed using the Screen for Child Anxiety Related Emotional Disorders (SCARED) (44). The SCARED is a 41-item self-report measure assessing symptoms across multiple anxiety domains, including generalized anxiety, separation anxiety, social anxiety, and school avoidance. Items were rated on a five-point Likert scale ranging from 0 (not true or hardly ever true) to 4 (very true or often true). Subscale and total scores were computed by summing relevant items. For the present study, the measure was completed by mothers regarding their children’s symptoms, consistent with prior research using maternal report (45). Internal consistency in the present sample was strong (*α* = .89).

### 3.5 Data analysis

Data were analyzed using partial least squares structural equation modeling (PLS-SEM) to examine the measurement and structural models (46). The analysis followed a two-step approach: (1) evaluation of the measurement model (reliability and validity) and (2) assessment of the structural model (path coefficients, effect sizes, predictive relevance, and mediation effects). Descriptive statistics and Pearson correlation coefficients were computed prior to SEM analyses. Bootstrapping procedures (5,000 resamples) were applied to estimate the significance of direct, indirect, and total effects. Multicollinearity was assessed using variance inflation factor (VIF) values. Missing data were present at low levels (generally <5% per variable). Because the number of valid observations varied across variables, variable-specific Ns are reported where applicable. For the PLS-SEM analyses, available observations were retained within the estimation procedure rather than applying listwise deletion to the full sample. Consequently, differences in variable-specific Ns reflect the availability of valid responses for individual measures, while the overall study sample comprised 343 participants.

### 3.6 Common method bias

Consistent with established recommendations for mitigating common method bias in self-report research (47), multiple procedural safeguards were implemented during data collection. Participants’ responses were collected anonymously to reduce social desirability and evaluation apprehension, and survey items were randomized to minimize order effects and pattern-based response tendencies. Following data collection, the presence of residual common method bias was assessed using Harman’s single-factor test (47). An unrotated exploratory factor analysis conducted on all self-report items indicated that the first unrotated factor accounted for well below the commonly accepted threshold of 40%, suggesting that common method bias is unlikely to have substantially influenced the results (47).

## 4 Results

### 4.1 Socio-demographic characteristics of the sample

A total of 343 Iranian mothers participated in the study. The mean age of participants was 42.12 years (SD = 7.03), and all participants had at least one child aged 5 to 10 years. The sample included mothers from both urban and conflict-affected regions, with the majority residing in Tehran (88.8%) and the remainder living in other provinces affected by the conflict (11.2%). More than half of the participants (58.0%) reported experiencing temporary or permanent displacement due to war-related events. Regarding marital status, the majority of participants were married (85.3%), while 9.1% were single (never married) and 5.6% were divorced. In terms of educational attainment, 43.4% held a bachelor’s degree, 44.8% held a master’s degree or higher, 10.5% had completed secondary education, and 1.4% had primary or middle school education. Occupational status indicated that 51.7% were housewives, 23.1% were employed full-time, 12.6% were employed part-time, 6.3% were students, and 6.3% were retired. Household composition varied, with family size ranging from one to seven members, and four-member households being the most common (40.0%). In terms of the number of children, 42.7% had two children, 28.7% had one child, 23.8% had three children, and 4.9% had four or more children. A detailed overview of socio-demographic characteristics is presented in **Table 1**.

**Table 1:** Socio-demographic characteristics of the study sample (N = 343).

Variable	Category	n	%	M	SD
Age (years)	—	—	—	42.12	7.03
Residence	Tehran	304	88.8	—	—
	Other provinces	39	11.2	—	—
Displacement status	Displaced	199	58.0	—	—
	Not displaced	144	42.0	—	—
Marital status	Married	292	85.3	—	—
	Single	31	9.1	—	—
	Divorced	19	5.6	—	—
Education	Primary/Top school	5	1.4	—	—
	Secondary school	36	10.5	—	—
	Bachelor’s degree	149	43.4	—	—
	Master’s or higher	153	44.8	—	—
Occupation	Housewife	178	51.7	—	—
	Full-time employed	79	23.1	—	—
	Part-time employed	43	12.6	—	—
	Student	22	6.3	—	—
	Retired	21	6.3	—	—
Number of children	1 child	99	28.7	—	—
	2 children	146	42.7	—	—
	3 children	82	23.8	—	—
	≥4 children	17	4.9	—	—

### 4.2 Correlation analysis

Prior to testing the structural model, bivariate correlations were computed for all study variables (**Table 2**). War-related stressors demonstrated significant positive correlations with both maternal anxiety and child anxiety. Threat appraisal was positively associated with war stressors, maternal anxiety, and child anxiety, whereas challenge appraisal exhibited significant negative correlations with these variables. Problem-focused coping was positively associated with challenge appraisal and negatively associated with threat appraisal. Emotion-focused coping demonstrated significant negative correlations with war stressors and maternal anxiety, while cognitive restructuring showed negative correlations with war stressors and child anxiety. Religious coping was negatively correlated with both child anxiety and maternal anxiety. Social support was negatively correlated with war stressors, maternal anxiety, and child anxiety, and positively correlated with challenge appraisal. All correlation coefficients were within acceptable ranges, and no evidence of multicollinearity was observed (48).

**Table 2:** Correlation studies. .

No.	Constructs	1	2	3	4	5	6	7	8	9	Mean	SD
1	WS	1									2.06	0.91
2	MA	0.50	1								3.73	0.81
3	CA	0.42	.389^**^	1							2.62	0.31
4	CHP	-0.47	-0.20^*^	-0.387^**^	1						1.33	0.58
5	TA	0.41	.307^**^	0.584^**^	-0.16^*^	1					1.60	0.68
6	PFC,	0.43	-.176^*^	-0.301^**^	0.49^**^	-.280^**^	1				2.66	0.81
7	EFC	-0.61	-.376^**^	-.310^**^	0.41^**^	-.233^**^	.496^**^	1			2.68	0.59
8	COR	-0.49	-.301^**^	-.345^**^	0.39^**^	-.376^**^	0.35^**^	0.248^**^	1		1.81	0.54
9	RL	-0.22^**^	-.221^**^	-0.37^**^	0.16^*^	-0.28^**^	0.79^**^	0.347^**^	0.169^*^	1	3.78	0.97
10	SS	-0.52	-0.53^**^	-0.49^**^	0.61^**^	-0.201^*^	0.320^**^	0.466^**^	0.163^*^	.427^**^	2.49	0.91

WS, War stressor; MA, Maternal anxiety; CA, Children anxiety; CHP, Challenge appraisal; TA, Threat appraisal; PFC, Problem-focused coping; EFC, Emotion-focused coping; COR, Cognitive restructuring; RL, Religiosity; SS, Social support; ^**^Correlation is significant at the 0.01 level (2-tailed).

#### 4.2.1 Measurement model assessment

The measurement model was evaluated by examining internal consistency reliability, convergent validity, and discriminant validity using PLS-SEM procedures.

##### 4.2.1.1 Reliability and convergent validity

Internal consistency reliability was assessed through Cronbach’s alpha, rho_A, and composite reliability (CR). As presented in **Table 3**, Cronbach’s alpha values ranged from 0.661 (children’s anxiety) to 0.913 (war stressor), indicating acceptable to excellent reliability. Although the alpha value for child anxiety was slightly below the conventional threshold of.70, its composite reliability (CR = 0.815) exceeded the recommended cutoff of.70, supporting adequate reliability despite the lower alpha value. All other constructs demonstrated satisfactory reliability, with CR values ranging from 0.842 to 0.931, exceeding the recommended threshold of.70. Rho_A values were also above.70 for all constructs, further confirming reliability.

**Table 3:** Reliability and convergent validity of the measurement model.

Dimension	α	rho_A	CR	AVE
WS	.913	.944	.926	.612
MA	.894	.896	.919	.656
CA	.661	.671	.815	.595
CHP	.907	.771	.900	.694
TA	.763	.780	.865	.683
PFC	.778	.779	.871	.694
EFC	.716	.717	.842	.640
COR	.749	.813	.842	.640
SS	.768	.850	.883	.792
RL	.908	.929	.931	.732

WS, War stressor; MA, Maternal anxiety; CA, Child anxiety; CHP, Challenge appraisal; TA, Threat appraisal; PFC, Problem-focused coping; EFC, Emotion-focused coping; COR, Cognitive restructuring; RL, Religiosity; SS, Social support.

Convergent validity was evaluated using the average variance extracted (AVE). All AVE values were above the recommended threshold of.50, ranging from 0.595 to 0.792, indicating that each construct explained more than 50% of the variance in its indicators. These results confirm adequate convergent validity of the measurement model.

##### 4.2.1.2 Discriminant validity

Discriminant validity of the measurement model was assessed using both the Fornell–Larcker criterion and the heterotrait–monotrait ratio (HTMT). As shown in **Table 4**, the square roots of the average variance extracted (AVE) for all constructs were greater than the corresponding inter-construct correlations, indicating satisfactory discriminant validity according to the Fornell–Larcker criterion. However, one relatively high correlation was observed between child anxiety and war stressors (*r* = .775), approaching the square root of the AVE for child anxiety (.816). Although the value remained below the threshold, indicating acceptable discriminant validity, this strong association suggests conceptual proximity between these constructs. Examination of HTMT ratios further supported discriminant validity, as all values were below the recommended cutoff of 0.85. The highest HTMT value was observed between challenge appraisal and child anxiety (HTMT = 0.690), which still falls within acceptable limits. To further strengthen construct distinctiveness, cognitive restructuring and threat appraisal were carefully examined as theoretically related yet empirically distinct variables within the extended transactional model. Overall, the results indicate that the measurement model demonstrates adequate discriminant validity, confirming that each construct captures a unique aspect of stress appraisal, coping processes, psychosocial resources (religiosity and social support), and anxiety outcomes among mothers and their children.

**Table 4:** Discriminant validity.

Fornell–Larcker Criterion												Heterotrait–Monotrait Ratio (HTMT)									
No.	Construct	1	2	3	4	5	6	7	8	9	10	1	2	3	4	5	6	7	8	9	10
1	CA	**.816**										—									
2	COR	–.344	**.809**									0.379	—								
3	EFC	–.361	.335	**.801**								0.421	0.386	—							
4	MA	.406	–.474	–.618	**.809**							0.425	0.541	0.746	—						
5	PFC	–.335	.141	.478	–.303	**.821**						0.364	0.169	0.596	0.307	—					
6	RL	–.141	.114	.472	–.289	.294	**.891**					0.144	0.124	0.628	0.317	0.411	—				
7	SS	–.089	.089	.294	–.062	.272	.740	**.890**				0.089	0.110	0.385	0.070	0.357	0.390	—			
8	TA	–.106	.023	.020	–.005	.005	–.020	.062	**.812**			0.093	0.167	0.060	0.081	0.092	0.057	0.064	—		
9	CHP	–.622	.342	.315	–.321	.377	.217	.199	.028	**.825**		0.690	0.462	0.426	0.382	0.452	0.308	0.240	0.072	—	
10	WS	.775	.264	.293	–.310	.289	.157	.047	.089	.402	**.832**	0.038	0.366	0.447	0.427	0.415	0.200	0.066	0.162	0.591	—

WS, War stressor; MA, Maternal anxiety; CA, Child anxiety; CHP, Challenge appraisal; TA, Threat appraisal; PFC, Problem-focused coping; EFC, Emotional-focused coping; COR, Cognitive reconstructing; RL, Religion; SS, Social support. Diagonal values (in bold) represent the square root of the average variance extracted (AVE) for each construct. Off-diagonal values represent the correlations between constructs. Discriminant validity is supported when diagonal values exceed the corresponding off-diagonal correlations.

#### 4.2.2 Structural model assessment

The structural model was assessed by examining collinearity (VIF), coefficient of determination (*R²*), predictive relevance (*Q²*), and model fit indices. Collinearity diagnostics indicated that all VIF values were below the recommended threshold of 5, with the highest values observed for maternal anxiety (VIF = 3.582) and war-related stressors (VIF = 2.611), confirming that multicollinearity did not pose a significant threat to the structural model. The model demonstrated strong explanatory power, with R² values of 0.72 for child anxiety and 0.801 for maternal anxiety, indicating that the model accounted for a substantial proportion of variance in these key outcomes. Moderate explanatory power was observed for child anxiety (*R²* = 0.395) and problem-focused coping (*R²* = 0.561), whereas weaker explanatory power was found for cognitive appraisal (*R²* = 0.097) and threat appraisal (*R²* = 0.008). Predictive relevance, assessed using the Stone–Geisser *Q²* statistic, was established for all endogenous constructs, with child anxiety (*Q²* = 0.670) and maternal anxiety (*Q²* = 0.199) demonstrating strong predictive relevance. Threat appraisal also yielded an acceptable *Q²* value (*Q²* = 0.152), confirming adequate predictive relevance across all constructs. Model fit indices indicated good model fit, with the saturated model SRMR = 0.079 and the estimated model SRMR = 0.068, both below the recommended threshold of 0.08. Additionally, normed fit index (NFI) values exceeded 0.90 for both models, further supporting acceptable model fit. Overall, these findings indicate that the structural model demonstrates adequate fit and requires no further refinement.

##### 4.2.2.1 Structural model

The findings showed that two of the three hypotheses were supported by significant associations at varying levels of significance (**Table 5**). War stressors were found to be significantly and positively associated with child anxiety (*β* = 0.60, *t* = 11.47, *SD* = 0.052, *f²* = 1.016, *p* <.001). Consequently, this finding confirmed H_2_. In contrast, war stressors were not significantly related to maternal anxiety (*β* = 0.014, *t* = 0.19, *SD* = 0.074, *f²* = 0.000, *p* = .910). Thus, the results rejected H_1_. The results also showed that maternal anxiety was significantly associated with child anxiety (*β* = 0.28, *t* = 2.42, *SD* = 0.116, *f²* = 0.036, *p* = .016). Therefore, the findings confirmed H_8_. According to the current research, the *R²* value was considerably high at 0.722 for children’s anxiety and 0.801 for maternal anxiety.

**Table 5:** Results of the path analysis (direct effects).

Hypothesized path	Path coefficient	*t*	*SD*	*f²*	*p*	Decision
H_1_: WS→MA	0.014	0.19	0.074	—	0.910	NS
H_2:_ WS→CA	0.600	11.47	0.052	1.016	<.001	S
H_8:_ MA→CA	0.280	2.42	0.116	0.036	0.016	S

WS, War stressor; MA, Maternal anxiety; CA, Child anxiety; IV, independent variable; DV, dependent variable; SD, standard deviation; *f²*, effect size. NS, Not supported; S, Supported; SD, Standard deviation, Standard deviation values are derived from the bootstrap procedure.

#### 4.2.3 Mediation test

The association between war-related stressors and maternal anxiety was significant in the direct model (*β* = 0.327, *t* = 3.31, *p* = .001), as displayed in **Table 6**. In the presence of threat appraisal, the relationship between war-related stressors and child anxiety remained significant (*β* = 0.179, *t* = 4.98, *p* <.001). However, the size of the standard regression weight decreased. The indirect effect via threat appraisal was not significant (*β* = –0.004, *t* = 0.62, *p* = .615); thus, the results rejected H_3_. With the inclusion of challenge appraisal as a mediator, the relationship between war-related stressors and maternal anxiety was significant (*β* = 0.147, *t* = 3.97, *p* <.001), and the indirect effect was significant (*β* = 0.147, *p* = .000); thus, the results confirmed H_4_. For problem-focused coping, the indirect effect on the relationship between war-related stressors and maternal anxiety was significant (*β* = 0.102, *p* = .029), confirming H_5_. Similarly, cognitive restructuring demonstrated a significant indirect effect (*β* = 0.100, *p* = .008), confirming H_6_. For emotion-focused coping, the indirect effect on the relationship between war-related stressors and child anxiety was significant (*β* = 0.168, *p* = .013), supporting H_7_. Overall, these findings indicate that mediation effects are partial rather than full, given that the direct effects of war-related stressors on both maternal and child anxiety remain significant. With the inclusion of these mediators, the *R²* values for maternal anxiety and child anxiety were 0.801 and 0.722, respectively.

**Table 6:** Mediating effect.

Hypothesized path	*β*	*t*	*P*	95% CI Bootstrap BC	
				LB	UB
WS→MA	.327	3.31	.001	.153	.51
WS→CA	.179	4.98	.000	.109	.24
H_3_: WS→TA→CA	-.004	.62	.615	-.017	.015
H_4_: WS→CHA→MA	.147	3.97	.000	.082	.213
H_5_: WS→PFC→MA	.102	2.19	.029	.032	.223
H_6_: WS→COR→MA	.100	2.65	.008	.009	.196
H_7_: WS→EFC→CA	.168	2.49	.013	.040	.184

WS, War stressor; MA, Maternal anxiety; CA, Child anxiety; TA, Threat appraisal; CHP, Challenge appraisal; PFC, Problem-focused coping; COR, Cognitive restructuring; EFC, Emotion-focused coping; β, standardized coefficient; t, t-value; p, significance level; CI, confidence interval; LB, lower bound; UB, upper bound.

#### 4.2.4 Moderation test

The interaction–moderation results revealed a significant association between the interaction of war-related stressors and religiosity on maternal anxiety (*β* = 0.109, *t* = 2.955, *p* = .003). Therefore, religiosity played a moderating role in the association between war-related stressors and maternal anxiety, and H_9_ was supported. The interaction effect of war-related stressors and social support on maternal anxiety was also significant (*β* = 0.086, *t* = 2.074, *p* = .039). Consequently, social support moderated the relationship between war-related stressors and maternal anxiety, confirming H_11_. In contrast, the interaction effect of war-related stressors and social support on child anxiety was not significant (*β* = 0.007, *t* = 0.172, *p* = .863). Thus, the results rejected H_10_.

## 5 Discussion

This study examined the relationships between war-related stressors, cognitive appraisal, coping strategies, and anxiety disorders among Iranian mothers and their children following a recent military conflict in the Middle East. The findings provide empirical support for several key pathways within the transactional model of stress and coping (6), while also revealing findings that contribute to further refinement of the model.

Contrary to the hypothesized direct effect, war-related stressors were not significantly associated with maternal anxiety (H_1_ was rejected). This finding does not align with prior research documenting strong associations between war exposure and maternal distress (44). Nevertheless, the significant indirect effects through challenge appraisal, problem-focused coping, and cognitive restructuring suggest that the relationship between war stressors and maternal anxiety is partially mediated by cognitive and coping processes. This underscores the central tenet of the transactional model that stressors influence outcomes through both direct exposure effects and appraisal-based mechanisms (1). In contrast, war-related stressors were directly and strongly associated with child anxiety (H_2_), consistent with studies documenting the profound psychological impact of war exposure on children (45). This differential pattern may reflect children’s greater vulnerability to objective stressors, as they have fewer developed coping resources and rely heavily on caregivers for emotional regulation (46).

The significant positive association between maternal anxiety and child anxiety (H_8_) aligns with the intergenerational transmission literature. This finding is consistent with studies showing that maternal psychological functioning influences children’s adjustment in war contexts (49, 50). It also supports recent work by Buchnik-Atzil et al. (2), who found that maternal anxiety positively predicted child emotional distress during war-related stress. However, it contrasts with research from Kosovar families 11 years post-war that found no such association (51), suggesting that transmission may be more pronounced in the immediate aftermath of conflict. The present findings also align with Karam et al. (52), who demonstrated the enduring impact of war-related anxiety on children and adolescents, and with Halevi et al. (53), who found that maternal stress physiology and synchronous parenting mediate the effects of war exposure on child psychopathology.

Regarding mediation, threat appraisal did not mediate the relationship between war stressors and child anxiety (H_3_). This null finding may indicate that child anxiety responses to war exposure are more directly driven by objective threat exposure rather than subjective appraisal processes. Alternatively, it may reflect the fact that mothers reported on both war exposure and child anxiety, potentially inflating direct associations. The significant positive indirect effect of war stressors on maternal anxiety through challenge appraisal (H_4_) supports theoretical work suggesting that viewing stressors as challenges fosters adaptive coping and positive outcomes (11, 54, 55). This finding highlights the protective function of challenge appraisals in mitigating distress.

The adaptive roles of problem-focused coping (H_5_) and cognitive restructuring (H_6_) as mediators align with extensive literature linking active, approach-oriented coping to better mental health outcomes (55). These findings suggest that mothers who engaged in active problem-solving and cognitive reframing experienced less anxiety despite high war exposure. Conversely, emotion-focused coping positively mediated the relationship between war stressors and child anxiety (H_7_), consistent with evidence that avoidance and disengagement strategies are maladaptive in trauma contexts (56, 57). This may reflect that when mothers rely on emotion-focused coping, they model maladaptive strategies for children or have fewer emotional resources to support their children’s regulation. These findings are further supported by Blikhar (58), who highlighted the role of parental attitudes and family upbringing styles in shaping children’s fear and anxiety responses.

The moderation findings revealed important protective roles for religiosity and social support. Religiosity weakened the positive association between war stressors and maternal anxiety (H_9_), consistent with research on religious coping as a meaning-making resource (59, 60). Social support similarly moderated this relationship (H_11_), supporting the buffering hypothesis (61) and aligning with Sippel et al. (62), who emphasized the critical role of social support in trauma-exposed families. However, social support did not moderate the relationship between war stressors and child anxiety (H_10_). This contrasts with Enav and Mayer (63), who found that parental reflective functioning moderated the relationship between war exposure and child distress. The null finding may reflect that social support operates differently for children, with factors such as maternal self-efficacy and parent-child communication playing more direct roles (4). Additionally, Quittner et al. (64) found that social support mediated rather than moderated parenting stress, suggesting that the role of social support may vary across contexts and outcomes. Ulmer-Yaniv et al. (65) further demonstrated that maternal immune and affiliative biomarkers, along with sensitive parenting, mediate the effects of chronic early trauma on child anxiety, highlighting the biological and relational mechanisms involved in intergenerational transmission.

## 6 Conclusion

Overall, the findings support an integrated model in which war-related stressors influence maternal anxiety indirectly through cognitive appraisal and coping processes, while directly impacting child anxiety. Importantly, the results do not support a primary intergenerational transmission pathway in the strict sense. Instead, they indicate a dual-pathway family stress model in which children are directly affected by war-related stressors, while maternal anxiety contributes as a secondary and partial influence. Maternal anxiety functions as a contributing factor within a broader family system of distress rather than the central transmission mechanism of child anxiety. This pattern is consistent with the strong direct effect of war-related stressors on child anxiety and the comparatively smaller but significant effect of maternal anxiety on child outcomes. Sociocultural resources such as religiosity and social support provide important protection for mothers, buffering the psychological impact of war-related stress exposure. These findings contribute to the growing body of literature on war-related trauma and family mental health by highlighting that post-conflict psychological outcomes in children are shaped by both direct exposure to traumatic events and indirect familial processes operating within a systemic framework of shared trauma. Finally, the study underscores the need for culturally sensitive interventions that address both child-centered trauma exposure and maternal mental health simultaneously, rather than assuming a unidirectional transmission from mother to child.

## 7 Implications

This study offers several practical implications for trauma and stress management interventions targeting war affected families. First, the significant mediating role of cognitive appraisal suggests that interventions designed to modify how individuals interpret stressful events may be particularly effective. Programs that help mothers and children reframe war related experiences from threats to challenges, such as cognitive behavioral interventions incorporating cognitive restructuring, could reduce anxiety symptoms, and practitioners should assess clients’ appraisal styles and target maladaptive threat appraisals as a key component of treatment. Second, the differential effects of coping strategies highlight the importance of promoting adaptive coping while reducing reliance on maladaptive emotion focused coping. Interventions should equip mothers with active coping skills, such as problem solving and cognitive reframing, while helping children reduce avoidance based strategies, partly by encouraging caregivers to model adaptive coping behaviors.

Third, the moderating effects of religiosity and social support indicate that these resources can serve as protective factors. Mental health programs should consider integrating culturally sensitive religious and spiritual practices, such as prayer, spiritual counseling, or engagement with religious leaders, and strengthening social support networks through family based interventions, peer support groups, and community mobilization, particularly in contexts where religion and community ties play a central role in daily life. Fourth, the significant association between maternal anxiety and child anxiety underscores the importance of family based approaches: interventions addressing maternal psychological wellbeing may have cascading benefits for children, and health care providers should screen mothers for anxiety and offer timely referrals. Finally, the strong explanatory power of the model (R² = 0.801 for maternal anxiety and R² = 0.722 for child anxiety) and the timing of the study, three months after the cessation of active conflict, highlight a critical window for early, multi-level intervention. Community based delivery models implemented through schools, health centers, and religious institutions may be particularly effective in reaching families who might not otherwise access formal mental health services, supporting a holistic approach that addresses cognitive appraisals, coping behaviors, social resources, and intergenerational dynamics rather than focusing solely on symptom reduction.

## 8 Limitations and future directions

Despite the theoretical and methodological contributions of this study, several limitations should be considered when interpreting the findings. First, the cross-sectional design limits the ability to draw causal conclusions among the study variables. Because data were collected at a single time point approximately three months following the military conflict, temporal ordering among war-related stressors, appraisal processes, coping strategies, and anxiety outcomes cannot be established. Accordingly, the observed association between maternal and child anxiety should not be interpreted as evidence of a directional or intergenerational transmission process but may instead reflect concurrent psychological responses to shared trauma exposure, emotional contagion, shared environmental threat, or potentially bidirectional influences. Future studies should employ longitudinal or prospective designs to better understand how stressors, appraisal processes, coping strategies, and anxiety develop and influence one another over time. Second, child anxiety was assessed through maternal reports rather than direct child self-reports. While parent reports are commonly used for younger children, they may be influenced by the mother’s own psychological state and perceptions. Future research should incorporate multiple sources of information, including child self-reports, teacher reports, or clinical assessments, to obtain a more comprehensive evaluation of children’s emotional functioning. Third, the sample primarily consisted of highly educated mothers living in urban areas. This may limit the generalizability of the results to other populations, such as mothers with lower educational levels, families living in rural settings, or other caregivers including fathers and extended family members. Future research should recruit more diverse samples across different socioeconomic backgrounds, geographic regions, and family roles to enhance the external validity of the findings. Third, the sample was predominantly composed of highly educated mothers residing in Tehran, which may limit the generalizability of the findings to mothers with lower educational attainment, different socioeconomic backgrounds, and those living in other geographic or rural settings. In the present sample, 43.4% of mothers held a bachelor’s degree and 44.8% held a master’s degree or higher, while only 11.9% had secondary education or less. Although stratified proportional sampling was used to improve representation across geographic location and displacement status, differences in educational and geographic composition remained. Future research should recruit more socioeconomically and educationally diverse samples across urban and rural settings and include fathers and other caregivers to strengthen external validity. Finally, some concerns related to discriminant validity were observed in the measurement model, particularly between religiosity and social support, as well as between emotion-focused coping and maternal anxiety. These patterns may indicate partial conceptual overlap among these constructs. Future studies should further refine measurement instruments and examine these relationships using alternative scales or mixed-method approaches to better distinguish related psychosocial factors.

## Data Availability

The raw data supporting the conclusions of this article will be made available by the authors, without undue reservation.
