# Supplementary material for: Understanding anxiety disorders among Iranian mothers and their children exposed to conflict using an extended transactional model of stress and coping
Source: Front Psychiatry. 2026 Oct 16;17:1839849. doi: 10.3389/fpsyt.2026.1839849 (PMC13720533; doi:10.3389/fpsyt.2026.1839849)
Supplement: Supplementary file 1 [file Table1.docx]

The questionnaires used in this study.

| **Constructs** | **Items** |
| --- | --- |
| War-Related Stressors | I experienced displacement or had to temporarily leave my usual home because of the conflict. |
|  | I was required to stay in a temporary or unfamiliar place because of the conflict. |
|  | My home or property was damaged or destroyed because of the conflict. |
|  | I experienced serious disruption to electricity, water, food, transportation, or other basic necessities because of the conflict. |
|  | I experienced difficulty accessing healthcare or medications because of the conflict. |
|  | I experienced disruption to my employment, household income, or financial security because of the conflict. |
|  | I experienced separation from close family members because of the conflict. |
|  | Someone close to me was injured because of the conflict. |
|  | Someone close to me was killed or seriously harmed because of the conflict. |
|  | I witnessed violence, destruction, explosions, or other frightening conflict-related events. |
|  | I directly experienced a situation in which I feared that I or a family member could be seriously injured or killed. |
|  | I experienced intense fear for the safety of my child or children during the conflict. |
|  | My child's education, childcare, daily routine, or social activities were disrupted because of the conflict. |
|  | I experienced persistent uncertainty about what would happen to me or my family during the conflict. |
|  | I experienced repeated exposure to frightening information about the conflict through television, social media, telephone communication, or other sources. |
| Threat appraisal | I felt this stressful event would have a negative impact on me. |
|  | I felt this stressful event would have a negative impact on my family. |
|  | I felt this stressful event would have a negative impact on my child/children. |
|  | I felt this stressful event would have a negative impact on my future. |
|  | I felt this stressful event would have a negative impact on my financial situation. |
|  | I felt this stressful event would have a negative impact on my daily life. |
| Challenge Appraisal | I felt I had the skills necessary to overcome the stressful event. |
|  | I felt I could grow from the stressful event. |
|  | I felt I could learn from the stressful event. |
|  | I felt I could handle the stressful event. |
|  | I felt I could overcome the stressful event. |
|  | I felt the stressful event would have a positive outcome. |
| Problem-Focused Coping | I've been concentrating my efforts on doing something about the situation I'm in. |
|  | I've been taking action to try to make the situation better. |
|  | I've been trying to come up with a strategy about what to do. |
|  | I've been thinking hard about what steps to take. |
| Cognitive Restructuring | I've been trying to see it in a different light, to make it seem more positive. |
|  | I've been looking for something good in what is happening. |
|  | I've been accepting the reality of the fact that it has happened. |
|  | I've been learning to live with it. |
| Emotion-Focused Coping | I've been saying things to let my unpleasant feelings escape. |
|  | I've been expressing my negative feelings. |
|  | I've been turning to work or other activities to take my mind off things. |
|  | I've been doing something to think about it less, such as going to movies, watching TV, reading, daydreaming, sleeping, or shopping. |
| Religiosity / Religious Coping | I prayed to God for protection for myself and my family. |
|  | I turned to God when I felt frightened or uncertain. |
|  | I recited or listened to verses from the Quran to help me cope with stress. |
|  | I engaged in Islamic religious practices more frequently because of the conflict. |
|  | My religious beliefs helped me find meaning in what was happening. |
|  | My faith gave me hope during difficult periods. |
|  | I sought guidance or comfort from a religious leader or knowledgeable religious person. |
|  | I relied on prayer when I felt unable to control the situation. |
|  | I believed that my faith could help me and my family endure the difficulties associated with the conflict. |
|  | Religious practices helped me feel emotionally supported during the conflict. |
| **Maternal Anxiety** | Feeling nervous, anxious, or on edge |
|  | Not being able to stop or control worrying |
|  | Worrying too much about different things |
|  | Trouble relaxing |
|  | Being so restless it is hard to sit still |
|  | Becoming easily annoyed or irritable |
|  | Feeling afraid as if something awful might happen |
| **Child Anxiety** | When my child feels frightened, it is hard for them to breathe |
|  | My child gets headaches when at school |
|  | My child doesn't like to be with unfamiliar people |
|  | My child gets scared if they sleep away from home |
|  | My child worries about being liked by others |
|  | When frightened, my child feels like passing out |
|  | My child is often nervous |
|  | My child follows me wherever I go |
|  | People say my child looks nervous |
|  | My child feels nervous around people they don't know well |
|  | My child gets stomachaches at school |
|  | When frightened, my child feels like going crazy |
|  | My child worries about sleeping alone |
|  | My child worries about being as good as others |
|  | When frightened, things don't feel real to my child |
|  | My child has nightmares about something bad happening to their parents |
|  | My child worries about going to school |
|  | When frightened, my child's heart beats fast |
|  | My child gets shaky when scared |
|  | My child has nightmares about something bad happening to them |
|  | My child worries about things working out for them |
|  | When frightened, my child sweats a lot |
|  | My child is a worrier |
|  | My child gets really frightened for no reason at all |
|  | My child is afraid to be alone in the house |
|  | It is hard for my child to talk to unfamiliar people |
|  | When frightened, my child feels like choking |
|  | People say my child worries too much |
|  | My child doesn't like to be away from their family |
|  | My child is afraid of having anxiety or panic attacks |
|  | My child worries something bad might happen to their parents |
|  | My child feels shy around unfamiliar people |
|  | My child worries about the future |
|  | When frightened, my child feels like vomiting |
|  | My child worries about how well they do things |
|  | My child is scared to go to school |
|  | My child worries about past events |
|  | When frightened, my child feels dizzy |
|  | My child feels nervous when others watch them do something |
|  | My child feels nervous in social situations (parties, gatherings) |
|  | My child is shy |
